# Editorial: B Cell Activation and Differentiation: New Perspectives on an Enduring Topic

**DOI:** 10.3389/fimmu.2021.797548

**Published:** 2021-11-09

**Authors:** Zhenming Xu, Mark R. Boothby, Jayanta Chaudhuri

**Affiliations:** ^1^ Department of Microbiology, Immunology and Molecular Genetics, The Joe R. and Teresa Lozano Long School of Medicine, University of Texas Health Science Center San Antonio, San Antonio, TX, United States; ^2^ Department of Pathology, Microbiology & Immunology, Department of Medicine, Vanderbilt University School of Medicine, Nashville, TN, United States; ^3^ Immunology Program, Memorial Sloan Kettering Cancer Center, New York, NY, United States

**Keywords:** B cell, germinal center, antibody response, autoimmunity, infection and vaccine

B lymphocytes, identified more than half a century ago, are crucial for sustained host immune responses to pathogens through differentiation into long-lived plasma cells and memory B cells, particularly those producing specific antibodies of switched immunoglobulin (Ig) isotypes (IgG, IgA, and IgE). Reflecting the diversity of microorganisms that they need to counter, B cells function as both innate and adaptive immune cells, with their activation driven or fine-tuned by signals from a multitude of immune receptors as well as conventional proteins such as integrins and nuclear receptors. Consequently, B cells significantly alter the network of signal transducers as well as transcription factors, thereby re-shaping their epigenome and transcriptome to instruct their activation, migration, proliferation, differentiation, and survival, particularly in the germinal center (GC) microenvironment. New insights have also been generated to understand how dysregulation of B cell responses leads to autoimmunity.

During the antibody response, B cells extensively interact with CD4^+^ pre-T follicular helper (pre-Tfh) cells at the T-B border and with fully developed Tfh cells in follicles. In this Research Topic issue, Wishnie et al. focus on recent studies showing how such interactions are exquisitely regulated by the affinity of B cell receptor (BCR) to the cognate antigen, leading to the selection of locations of B cell responses, i.e., either in extrafollicular areas or within GCs. They also describe the propensity of B cells to differentiate into antibody-secreting cells (ASCs) and memory B cells if expressing high- and low-affinity BCRs, respectively, likely influenced by the location of B cell activation. In addition to dictating the duration and quality of interactions with Tfh cells, BCRs, upon crosslinking, trigger signaling pathways, such as those activating MAPK and NF-κB. These can also be activated by signals from CD40, as engaged by Tfh cell-expressed CD154, B-cell activating factor receptor (BAFF-R), also a TNF family receptor, and toll-like receptors (TLRs), which are innate receptors recognizing molecular patterns. By focusing on recent advances on the crosstalk between BCR signaling with CD40, BAFF-R, and TLR signaling, Chen and Wang discuss how such crosstalk imposes a checkpoint in class switch recombination (CSR) at the immunoglobulin heavy chain (*Igh*) gene locus and antibody responses as well as in breaking the B cell tolerance. Besides the well-studied BCR, CD40 and TLR signaling, other receptors and intracellular signaling pathways have been shown by new studies to influence B cell development and differentiation. For example, Garis and Garrett-Sinha cover how the Notch signaling, an evolutionarily conserved pathway known to play a key role in hematopoietic stem cell maintenance and in specification of T lineage cells, also regulates B cells, including B lymphocyte lineage commitment, specification of marginal zone type B cells, and mature B cell activation and differentiation during immune responses. Sun et al. describe the impact of the Wnt signaling, a fundamental pathway involved in many aspects of biological systems, on B cell differentiation. They also contend that such impact is modulated by single-stranded oligonucleotides that target sclerostin, a Wnt antagonist.

The movement of B cells along the T:B border precedes the full-blown GC reaction. Ishihara et al. investigate the role of Rap1 small GTPase in this process by generating mice with B cell-specific knockout in both Rap1 isoforms, Rap1a and Rap1b. B cells from such double knockout mice are impaired in moving along a gradient of chemoattractants known to be critical for their localization in the follicles, leading to the defective GC formation, and antibody responses to immunization – B1a cell development is also defective in such mice. In addition to interacting with Tfh cells through CD40:CD154 engagement for their activation, B cells are heavily dependent on IL-21, the hallmark cytokine of Tfh cells, to sustain their GC reaction and differentiate into ASCs. In a research article, Wang et al. show that GC B cells, paradoxically, are sensitized by IL-21 to activate caspase 9 in the mitochondria-dependent intrinsic apoptosis pathway. Such caspase 9 activation would be contained if caspase 8 of the extrinsic apoptosis pathway remains inhibited by cFLIP to prevent amplification of the entire caspase network and irreversible cell death. Lack of linear ubiquitin assembly complex (LUBAC) results in cFLIP degradation and IL-21-induced apoptosis in CD40-activated B cells *in vitro* as well as GC B cell death *in vivo*, leading to defective antibody affinity maturation and the T-dependent antibody response. In an independent study, Wright et al. discover that BIM, a pro-apoptotic BCL2 family member, mediates IL-21-induced B cell apoptosis. Also, B cell-specific deficiency in BIM leads to uncontrolled expansion of B cells, production of a wide array of autoantibodies and tissue infiltration of lymphocytes, indicating that GC B cell apoptosis is checkpoint of autoimmunity, including lupus. Interestingly, the defective apoptosis of BIM-lacking B cells can be ameliorated by knockout of the BTK tyrosine kinase, suggesting a new signal transduction pathway operating in B cells, including those that have broken the tolerance. This adds to the role of PI3K signaling, which coordinates various signaling molecules involved in B cell development and activation, and IFNγ signaling, which is known to be elevated in both systemic lupus erythematosus (SLE) patients and mouse models of lupus, as reviewed by Bacalao and Satterthwaite. These authors also summarize recent data on how IFNγR signaling mediates the development of autoreactive GCs and autoantibody responses in murine lupus, particularly the enhancement of chromatin accessibility at potential transcription factor-targeting sites, alterations in DNA methylation, and acquisition of new histone acetylation that can be regulated by histone deacetylase inhibitors, as potential lupus therapeutics. The epigenetic regulation of B cell in immunity and SLE is further extended to microRNAs (miRNAs), which mediate genetic regulation at the post-transcriptional level. Given the relatively extensive literature on miRNAs in B cells, Schell and Rahman highlight miRNAs with confirmed functions in mouse models and provide a perspective on the areas of future studies and the potential of miRNA-centric therapeutics in SLE. Built on the observation that the frequency of memory B cells is increased in lupus mouse models and their previous seminal findings that autophagy specifically and critically maintains memory B cells, Jang et al. explore the role of memory B cell responses in lupus pathogenesis triggered by pristane injection. B cell-specific deletion of Atg7, which is essential for autophagy, leads to loss of autoreactive memory B cells, much reduced autoantibody production in pristane-treated mice, and attenuated development of glomerulonephritis and pulmonary inflammation. Importantly, autoantibody production in such knockout mice can be rescued by memory B cells isolated from pristane-injected wild-type mice, showing that autophagy is a new therapeutic target for lupus.

In work directed towards translating laboratory investigations into the understanding of human B cell immunology, Joosse et al. report the development of a new throughput screening pipeline to analyze the repertoire of autoreactive B cells in autoimmune patients. This pipeline entails phenotypical identification of different B cell subsets, expansion of such B cells *in vitro* and their differentiation into ASCs, and identification of autoantigen-specific B cells through autoantibody-specific ELISA. This robust tool allows the investigators to use a small amount of cryo-preserved peripheral blood mononuclear cells (PBMCs) to detect insulin-binding memory B cells in pre-symptomatic type 1 diabetes donors. Using naïve B cells, GC centroblasts, GC centrocytes, memory B cells, and plasma cells purified from human tonsils and a label-free LC-MS/MS approach, Díez et al. profile, for the first time, the proteome of human B cells at distinct differentiation stages and show that, despite the considerable overlap of their proteome, these B cells express factors associated with regulation of metabolic programming to instruct the transition between differentiation stages. These tools will likely be adapted by other investigators in the field to advance their own research on human B cells in different pathophysiological contexts. For example, Upasani et al. address the susceptibility of B cells, in addition to widely studied dendritic cells and macrophages, to acute infection by dengue virus (DENV) in a cohort of 60 Cambodian children, likely by using CD300a, a phosphatidylserine receptor, as the entry receptor. These findings are consistent with the observation that human B cells can support the replication of lab-adapted and patient-derived DENV strains *in vitro*, leading to the release of live viruses into the supernatant, but no apparent antibody-dependent enhancement effects. Direct DENV infection causes human B cells to proliferate *in vivo* and differentiate into plasma cells *in vitro*. Like DENV, malaria is a global burden of infectious disease and in urgent need of an effective vaccine against the *Plasmodium* parasite. As natural infection elicits a robust immune response against the blood stage of the parasite to provide protection against malaria, Gonzales et al. reason that a full understanding of the mechanisms, acquisition and maintenance of naturally acquired immunity would pave the way for the development of a potential vaccine against the blood stage of *Plasmodium falciparum*. This topic is especially timely in light of the recent field-work success with the RTS,S vaccine and its protection of children from lethal *falciparum* malaria. Like all FDA-approved vaccines, new malaria vaccine candidates would elicit highly specific/neutralizing IgG, and possibly IgM, antibodies.

Altogether, these primary research and review articles have made this Research Topic a collection of new insights and perspectives on B cell biology. We are grateful to all the authors for their efforts and wish them success in continually pushing their respective fields forward.

## Author Contributions

All authors listed have made a substantial, direct, and intellectual contribution to the work and approved it for publication.

## Funding

This work is supported by NIH/NIAID AI 153506 (to ZX) and Departmental funds of Pathology-Microbiology-Immunology Department, Vanderbilt University Medical Center (to MB).

## Conflict of Interest

The authors declare that the research was conducted in the absence of any commercial or financial relationships that could be construed as a potential conflict of interest.

## Publisher’s Note

All claims expressed in this article are solely those of the authors and do not necessarily represent those of their affiliated organizations, or those of the publisher, the editors and the reviewers. Any product that may be evaluated in this article, or claim that may be made by its manufacturer, is not guaranteed or endorsed by the publisher.

